# Patterns of infectious complications and their implication on health system costs after esophagectomy for esophageal cancer: Real-world data from three European centers

**DOI:** 10.1007/s00423-025-03709-5

**Published:** 2025-04-22

**Authors:** Anna Lucia Ledda, Ignazio Tarantino, Sabine Schiefer, Ulrich Ronellenfitsch, Artur Rebelo, Carsten Sekulla, Henrik Nienhüser, Christoph Michalski, Bruno Schmied, Jörg Kleeff, Johannes Klose

**Affiliations:** 1 Department of Visceral, Vascular and Endocrine Surgery, Martin-Luther-University Halle-Wittenberg, University Medical Center Halle, Ernst-Grube-Str. 40, 06120 Halle, Germany; 2 Department of General, Endocrine and Transplantation Surgery, Kantonsspital St. Gallen, VisceralSt. Gallen, Switzerland; 3 Department of General, Visceral and Transplant Surgery, University Hospital of Heidelberg, Heidelberg, Germany

**Keywords:** Esophageal cancer, Infectious complications, Microbiota, Health costs

## Abstract

**Purpose:**

Infectious complications occur frequently after esophagectomy leading to prolonged hospital stay and increased costs. This study aimed to analyze the pattern of infectious complications, the spectrum of associated microbiota, and its impact on health system costs in patients who underwent esophagectomy for esophageal cancer.

**Methods:**

All patients undergoing curative resection for histologically confirmed esophageal cancer between January 2017 and August 2022 were included. Patients’ survival was analyzed by Kaplan–Meier estimate. Contingency tables were applied to assess the association between microbiota and the occurrence of infectious complications and their impact on patients’ survival.

**Results:**

Four hundred forty-one patients who received a R0 resection for esophageal cancer were identified. Infectious complications occurred in 153 patients (34.7%). Pneumonia was the most frequent complication (28.8%) followed by anastomotic leakage (25.4%). Enterococcus and Candida species were the dominant microbiota associated with infectious complications (Candida species: OR 7.34, 95% CI 2.38–22.67) and anastomotic leakage (Enterococcus species: OR 6,15, 95% CI 1,51–24,99; Candida species: OR 7.14, 95% CI 2.48–20.56). Intensive care unit stay (mean 14.3 vs. 4.9 days, *p* < 0.001) and total hospital stay (mean 34.1 vs. 18.8 days, *p* < 0.001) were significantly longer in patients with infectious complications. Total health system costs (44.084 € vs. 25.907 €) increased after the occurrence of infectious complications.

**Conclusion:**

Infectious complications after esophagectomy are predominantly associated with the presence of Enterococcus and Candida species, leading to increased health system costs. Preventive antibiotic and antimycotic treatment might result in reduction of infectious complications and lower health system costs.

**Supplementary Information:**

The online version contains supplementary material available at 10.1007/s00423-025-03709-5.

## Introduction

Approximately 10 new cases of esophageal cancer per 100.000 inhabitants per year in Western Countries can be observed [1]. Histologically, squamous cell carcinoma in the upper part of the esophagus has to be distinguished from adenocarcinoma, predominantly located in the distal part of the tube. Treatment is based on multimodal therapy, depending on histological subtype and staging. Neo-adjuvant chemo- or radio-chemotherapy will be applied followed by surgery [2]. Esophageal resection with intrathoracic anastomosis (Ivor Lewis procedure) is the surgical treatment of choice. However, this operation is associated with high rates of postoperative mortality and morbidity [3]. Infectious complications including pneumonia, anastomotic leakage with mediastinitis and pleural empyema, and surgical site infections are the most frequent reasons for prolonged hospitalization and even death after surgery. Pneumonia is the most frequent complication after surgery, followed by anastomotic leakage [4–6]. Both conditions have to be treated with at least antibiotic and antimycotic treatment and in case of anastomotic leakage interventional therapy or even surgery. Of note, cancer patients with immunosuppression due to the underlying disease and eventual chemo- or radiotherapy are at an increased risk to develop infectious complications.

It is well acknowledged that microbiota from the upper gastrointestinal tract are associated with infectious complications after esophagectomy [7]. Strategies to reduce the burden of this source of pathogens, are however not commonly used.

The aim of the present study was to investigate the pattern of infectious complications after esophagectomy. Furthermore, the pattern of microbiota was correlated with the occurrence of infectious complications and its impact on auxiliary costs due to prolonged hospital stay was calculated.

## Material and methods

### Data source and cohort definition

Data from all patients with histologically proven squamous cell carcinoma (SCC) or adenocarcinoma of the esophagus who received esophageal resection with intrathoracic anastomosis (Ivor Lewis procedure) at University Hospital Halle (Saale), Germany, University Hospital of Heidelberg, Germany or Kantonsspital St. Gallen, Switzerland, were included in this retrospective cohort study. Patients with cervical anastomosis or those who did not receive an esophageal anastomosis were excluded.

### Data collection, definitions, and follow-up

All patients undergoing esophageal resection for esophageal cancer between January 2017 and August 2022 provided informed consent prior to the operation. Independent ethics committees at the University Hospital Halle (Saale), University Hospital of Heidelberg and Kantonsspital St. Gallen approved the present study (approval no. 2019–037, University Hospital of Halle). Defined clinical data were collected from the medical charts and registered in an electronic database. The data included age, gender, American Society of Anesthesiologists (ASA) score, the presence of reflux disease, Barrett Dysplasia, smoking and alcohol habits, histological subtype (SCC vs. adenocarcinoma), the administration of neoadjuvant chemotherapy, type of operation, and UICC stage (8 th edition).

All operations were performed using highly standardized procedures and were either completed or supervised by experienced upper gastrointestinal surgeons. Independently of the surgical technique – i.e. open, laparoscopically or robot-assisted, the surgical concept of radical two-field lymphadenectomy and formation of a gastro-esophageal anastomosis by stapling technique was followed. In detail, after mobilization of the stomach, D2 lymphadenectomy was performed. Then, the gastric conduit was created using 60 mm staplers. After completion of the abdominal part, thoracic operation was started. After dissection of the azygos vein, the esophagus was mobilized. Intrathoracal lympadenectomy included resection of the “mesoesophagus” including infracarinal lymph node dissection. In case of an open approach, thoracotomy was performed and the eosphagus was devided and the circular stapling device was inserted into the remaining esophagus and the gastric conduit, respectively. During minimally-invasive surgery, the esophgus was devided before mini-thoracotomy. After stapling of the anastomosis, the conduit was formatted using a 60 mm linear stapler. Operation was terminated after routine intrathoracic drain placement.

Postoperatively, a CT scan or endoscopy was only performed upon suspicious drain content and/or inadequate elevated infectious parameters.

Complications after esophagectomy were classified according to the Esophagectomy Complications Consensus Group (ECCG) [8]. Although anastomotic leakage rather leads to secondary infections, we included it into the broader term of postoperative infectious complication.

Follow-up was performed according to the national guidelines. Neoadjuvant and adjuvant chemotherapy was administered based on the recommendation of the local multidisciplinary tumor board.

### Statistical analysis

Continuous data are expressed as arithmetic mean and standard deviation (SD) or median and 95% confidence interval (95% CI). Student’s two-sided t-tests, Kruskal–Wallis tests, and Mann–Whitney U-tests were used to compare, as appropriate. Categorical data are presented as absolute numbers and relative frequencies and were compared using the χ^2^ test. Likelihood ratio tests were used for regression analysis. Kaplan–Meier estimation and Cox regression analysis were performed for survival analysis. Relevant risk factors for the occurrence of infectious complications and survival were identified with Analysis of Variances (ANOVA), and univariate Cox regression, as appropriate.

Health system cost analysis for German and Swiss patients was performed based on the specific diagnosis-related-group (DRG) register of the respective hospital. The data was gathered through the local hospital administration. We stratified the data by occurrence of infectious complications (including pneumonia, surgical site infection, catheter infection) and anastomotic leakage. We were able to compare the groups with the respective patients in whom these complications did not occur.

Calculations were performed using IBM SPSS Version 28–29 (IBM, Armonk, NY, USA). A *p* value < 0.05 was considered statistically significant.

## Results

### Patient characteristics

**Fig. 1:**
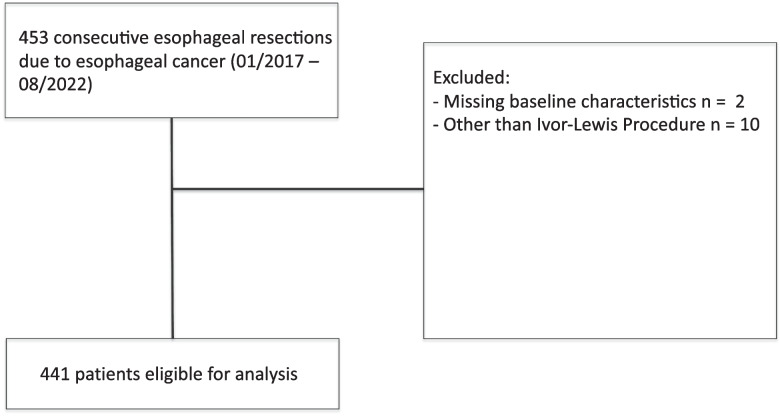
Flow chart of patient inclusion

During the study period, a total of 453 patients who underwent resection for esophageal cancer with intrathoracic gastro-esophageal anastomosis were identified. After excluding twelve patients due to missing data or wrong procedure, 441 patients remained for further analysis (Fig. 1). The majority of the patients were men and the mean age was 63 years. The baseline characteristics of all patients are depicted in Table 1. A total of 262 patients (59.4%) underwent open surgery. In 41 patients (9.3%), the abdominal part of the procedure was performed using a minimally invasive (laparoscopic) approach. In 54 patients (12.2%), both the abdominal and thoracic parts were performed laparoscopically. In 84 patients (19.0%), the procedure was performed robot-assisted (RAMIE). The mean operative time was 319.3 min (± 78.48 min). We observed a clear trend towards minimally-invasive surgery over the last years. Mean total hospital stay was 24.1 days and mean stay on the intensive care unit (ICU) was 8.1 days.

**Table 1:** Baseline characteristics for patients undergoing esophageal resection within the study period

	Patient characteristics (*n* = 441)	%
Age (years, mean)	63,4	
Gender		
male	363	82.3
female	78	17.7
ASA score		
I	3	0.7
II	162	36.7
III	268	60.8
IV	8	1.8
Reflux disease		
Yes	156	35.4
No	285	64.6
Barrett dysplasia		
Yes	92	20.9
No	346	78.5
unknown	3	0.7
Smoking		
Yes	216	49.0
No	225	51.0
Alcohol abuse		
Yes	93	21.1
No	348	78.9
Histology		
SCC	74	16.8
Adeno	367	83.2
Neoadjuvant Therapy		
Yes	372	84.4
No	68	15.4
Unknown	1	0.2
UICC stage		
0	66	16.7
I	212	54.1
II	47	12.0
III	53	13.4
IV	10	2.5
Unknown	6	1.3
Type of resection		
Open	262	59.5
Minimally-invasive	179	40.5
Infectious complications		
Yes	153	34.7
No	286	64.9
Catheter infection	18	2.9
Superficial surgical site infection	15	3.1
Pneumonia	127	28.8
Mediastinitis	17	3.9
Anastomotic leakage	112	25.4
N (%)		

*ASA*, American Society of Anaesthesiologists score; *SCC*, Squamous cell carcinoma; *UICC*, Union for international cancer control

One hundred and fifty-three patients (34.7%) developed infectious complications after esophagectomy. Pneumonia was the most common complication (28.8% of all patients) followed by anastomotic leakage (25.4%). The remaining infectious complications are listed in Table 1.

A total of 178 patients received antibiotic treatment only and 74 patients were treated with antibiotics and antimycotics. In 25 patients antibiotic treatment was continued after surgery prophylactically. Beta lactam antibiotics, i.e. Carbapenems were the most common administered drug class (*n* = 51), followed by Piperacillin/Tazobactame (*n* = 39), and Vancomycin (*n* = 28). Among the antimycotic drugs, Fluconazole was most frequently used (*n* = 48). The distribution pattern of the different antibiotic and antimycotic agents administered to the patients with infectious complications are shown in Table 2.

**Table 2:** Pattern of antibiotic and antimycotic treatment in patients with infectious complications

Antibiotic treatment	Treated patients (n)
Piperacillin/Tazobactame	39
Meropenem	34
Vancomycin	28
Imipenem	17
Metronidazole	12
Ciprofloxacin	10
Ceftriaxone	9
Moxifloxacin	7
Linezolide	5
Clarithromycin	3
Flucloxacillin	3
Erythromycin	2
Ceftazidime	2
Tigecycline	2
Fosfomycin	1
Daptomycin	1
Amoxicillin/Clavulanic acid	1
SZM/Trimethoprim	1
Cefepime	1
Antimycotic treatment	Treated patients (n)
Fluconazole	48
Caspofungin	22
Anidulafungin	3
Voriconazole	1

Out of the hundred twelve patients with anastomotic leakage, twelve patients (10,7%) developed Type III leakage: the anastomosis was oversewn in five patients, newly constructed in one patient, and resection of the anastomosis and cervical esophagostomy had to be done in the other six patients. Type II anastomotic leakage in the remaining one hundred patients (89.3%) was treated interventionally by EndoVac (76,8%), stent (8,9%) or combined EndoVac and stent placement (2,6%). Three patients (2,6%) were treated with a simultaneous CT-guided drain.

### Distribution of microbiota in different patient samples

To gain precise knowledge about the microbiota located in the different samples obtained in the cohort, we retrospectively analyzed microbiological reports. There was a wide spectrum of microbiota found in patients with infectious complications after esophagectomy. Enterococcus species were the predominant type of bacteria found in all sources of materials. Wound swab, intraabdominal swab, pleural effusion, drainage fluid, tracheal secretion, and bronchoalveolar lavage revealed likewise Enterococcus faecalis, faecium, and durans. Klebsiella and Pseudomonas species were also repeatedly found in all analyzed samples. Other bacteria including staphylococcus species and further enterobacteria were detected less frequently. All bacteria found are listed in Supplementary Tables S1 to S6.

We further analyzed the presence of multi-resistant lines among the microbial spectrum. Multi-resistant Gram-negative (MRGN) bacteria were found in one patient (0.6%). Bacteria producing extended-spectrum beta-lactamases (ESBL) were detected in ten patients (5.5%) and Vancomycin-resistant enterococci (VRE) were found in six patients (3.3%). In two patients (1.1%), Methicillin-resistant Staphylococcus aureus was detected.

Additionally, several Candida species were detected in the analyzed samples. Followed by Candida albicans, we also found Candida tropicalis and glabrata continuously. All fungi analyzed and their distribution among the different samples are shown in Supplementary Tables S1 to S6.

Enterococcus and Candida species were the dominant microbiota associated with infectious complications (Candida species: OR 7.34, 95% CI 2.38–22.67) and anastomotic leakage (Enterococcus species: OR 6,15, 95% CI 1,51–24,99; Candida species: OR 7.14, 95% CI 2.48–20.56). This correlation was independent of the source of the microbiota.

### Length of intensive care unit stay among patients with or without infectious complications

**Fig. 2:**
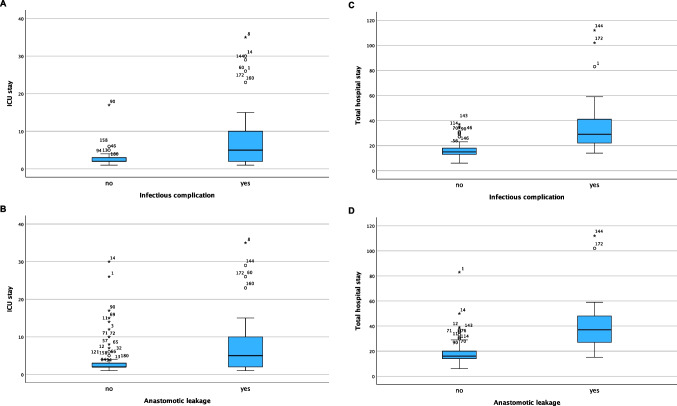
Length of intensive care unit (ICU) and total hospital stay in days depending on the occurrence of infectious complications (A + C) and anastomotic leakage (B + D)

Patients who developed infectious complications stayed significantly longer on intensive care unit (ICU) compared to patients without postoperative infections. As demonstrated in Fig. 2A, mean stay on ICU of patients with infectious complications was significantly longer (14 ± 18 days) compared to patients without complications (5 ± 5 days; *p* < 0.001).

When only looking at patients who developed anastomotic leakage, mean stay on the ICU was 17.61 (± 20.29) days compared to 5.1 (± 5.1) days for patients without anastomotic leakage, *p* < 0.001 (Fig. 2B).

Total hospital stay was also longer in patients with infectious complications (mean 34.1 ± 21.16 days) compared to those without infectious complications (mean 18.80 ± 9.50 days; *p* < 0.001, Fig. 2C).

For patients with anastomotic leakage, hospital stay was significantly longer (mean 40.28 ± 21.51 days) compared to patients without anastomotic leakage (mean 18.55 ± 8.92 days; *p* < 0.001, Fig. 2D).

We further performed univariate analysis to find variables associated with the occurrence of infectious complications. Our analysis (ANOVA) revealed that the only significant factor associated with the occurrence of infectious complications was an ASA Score > 3 (*p* = 0.012, see Supplementary Table S7.

### Patients’ survival depending on the occurrence of infectious complications

**Fig. 3:**
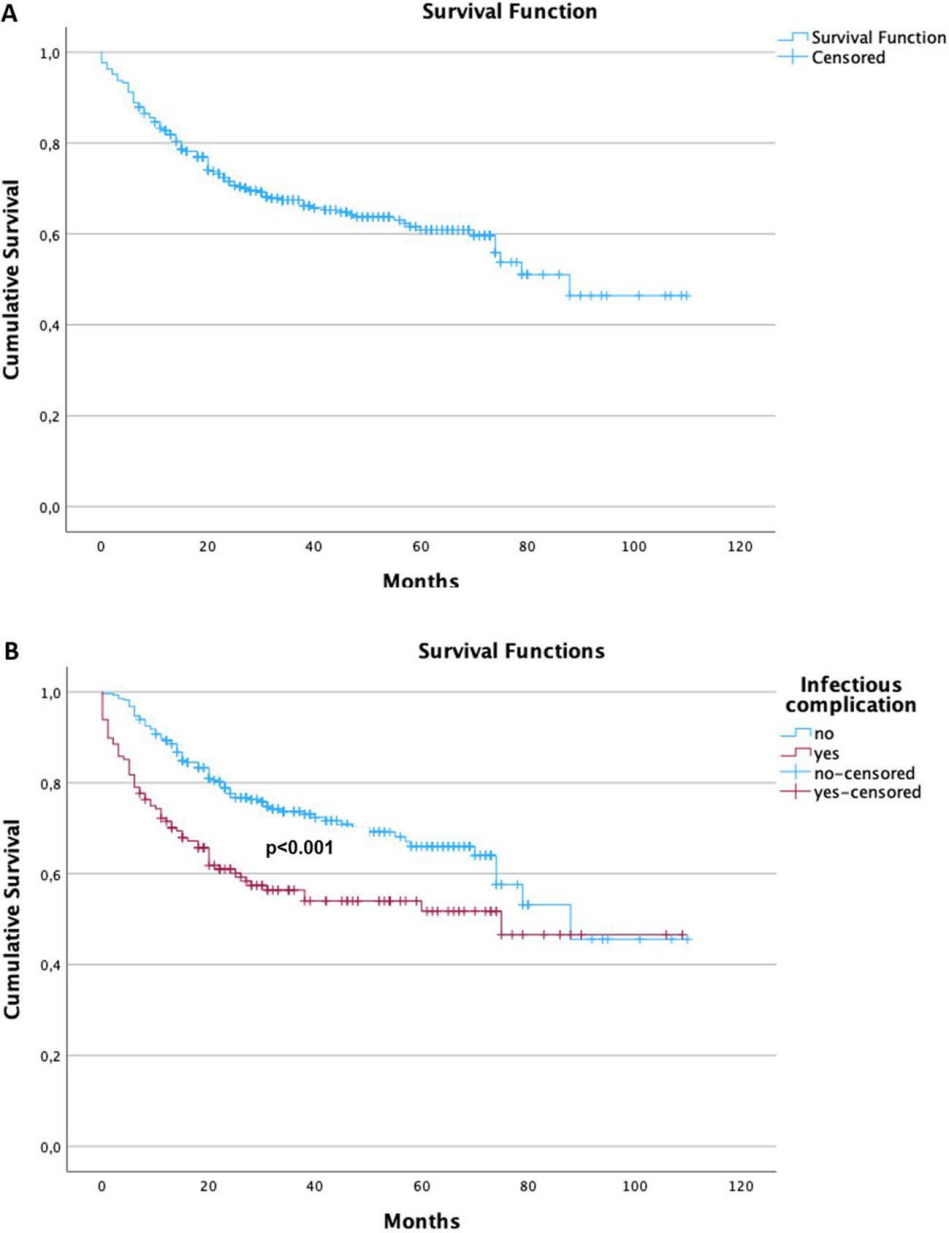
Kaplan Meier estimates for (**A**) overall survival and (**B**) stratified for the occurrence of infectious complications after esophagectomy

Patients’ survival after esophagectomy is depicted in Fig. 3A. 5-year overall survival was 60.9%.

After stratifying for the occurrence of infectious complications 5-year overall survival for patients with infectious complications was 51.7% compared to 66.0% for patients without infectious complications (*p* < 0.001). Figure 3B.

### Auxiliary health system costs due to infectious complications

Finally, we aimed to analyze auxiliary health system costs dependent on the occurrence of infectious complications. Therefore, we examined the hospital revenue for each patient within the study population. We distinguished between the German and the Swiss health care system given that compensation of health care services differs between both countries.

In Germany, mean revenue for esophageal resection for all patients was 32.735,87 Euros. A regular postoperative course after esophagectomy resulted in a mean revenue of 25.907,22 Euros compared to 44.084,12 Euros for a complicated course including the presence of infectious complications. The mean additional revenue for a complicated postoperative course was 18.176,90 Euros. After stratifying for the two most common infectious complications, namely pneumonia and anastomotic leakage, the revenue rose from 25.907,22 Euros to 45.445,41 and 48.495,83 Euros, respectively.

In Switzerland, the. mean revenue for all esophageal resections performed during the study period was 72.459,49 Swiss Francs. The mean revenue for esophageal resection without complications was 59.521,61 Swiss Francs. In contrast, the mean revenue in case of infectious complications was 96.394,57 Swiss Francs. Accordingly, mean difference in revenue was 36.872,96 Swiss Francs.

**Fig. 4:**
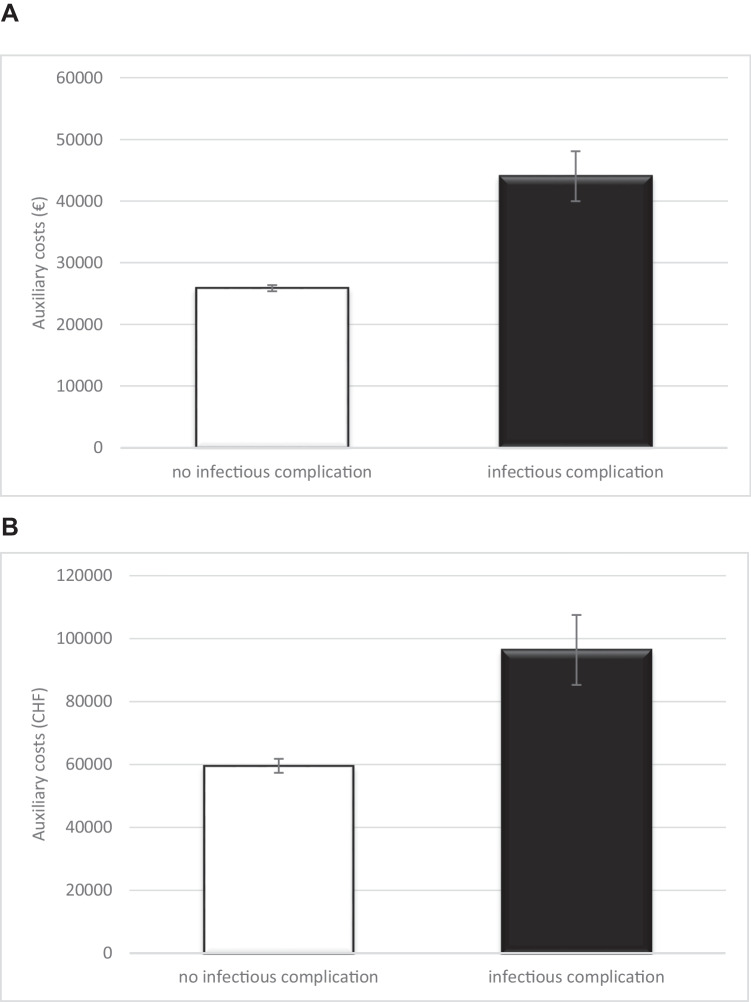
Auxiliary health care costs in patients suffering from infectious complications after esophagectomy in (**A**) Euros (Germany) and (**B**) Swiss Francs (Switzerland)

Auxiliary health costs for all patients are summarized in Fig. 4.

## Discussion

In the present investigation, we analyzed the clinical course of a cohort of all consecutive esophageal cancer patients who underwent Ivor-Lewis resection at three independent European institutions. We are able to demonstrate that infectious complications after esophagectomy occur in almost 35% of all patients. Enterococcus species were the most common bacterial source but no increase of multi-resistant pathogens was observed. Furthermore, our analysis confirmed that the presence of infectious complications was associated with prolonged ICU and total hospital stay. By analyzing hospital revenues for esophageal resection, we show the burden on health system costs associated with the occurrence of infectious complications. Our data suggest that profound strategies to avoid infectious complications have to be implemented to improve patients’ outcome and reduce economic health care burden.

For UICC stage I to III esophageal cancer, esophageal resection with intrathoracic anastomosis (Ivor-Lewis procedure) represents the gold standard of treatment. However, postoperative complications are frequent and result in increased postoperative morbidity and mortality. Pneumonia occurs in almost 40% of all patients undergoing esophagectomy [4]. This is roughly comparable with the results of our study, in which 28.8% of the patients developed postoperative pneumonia [9].

Anastomotic leakage occurred in 25.4% of patients in this study cohort. This is comparable with existing data in the literature describing rates of anastomotic leakage up to 25% [5, 6]. Almost 90% of the leakages in our cohort were successfully treated by endoscopy without re-operation.

Multivariate analysis revealed that in our cohort only an ASA score > 3 was associated with the occurrence of infectious complications after esophagectomy. Previous data indicate that malnutrition and sarcopenia are associated with a complicated postoperative course after esophagectomy [10, 11]. Given that we did not include those variables into the analysis, our data show that even patients with concomitant underlying diseases and mediocre general health status are under increased risk developing infectious complications after esophageal surgery.

The occurrence of infectious complications resulted in additional health system costs measured as the payments to hospitals in the presence of the diagnosis-related groups system compared to the normal course of treatment. This observation was equivalently noticed in all three centers analyzed and consequently relevant in two different European health care systems. Although an increased revenue was gained for the hospital upon the occurrence of infectious complications after esophagectomy, this is vice versa associated with increased costs for the hospital, which could however not be measured in the present study. Likewise, complications also cause indirect costs for the patient, which are difficult to quantify. Löfgren and co-workers have previously described the impact of increasing costs after esophageal resection caused by the occurrence of infectious complications in Sweden [12]. In line with our data from two other European countries, costs are increased after an aberrant course following esophagectomy, especially after the occurrence of infectious complications. Similar data are available from the Dutch Upper GI Cancer Group [13]. An analysis from the SEER-database by Jiang et al. further revealed an increased 90-day cost in patients who suffered complications after esophagectomy [14]. These observations raise the necessity of strategies to prevent infectious complications. In this regard, growing evidence suggests a pivotal role for microbiota in the esophagus as a driver for anastomotic leakage and other infectious complications after esophagectomy [9, 15]. The main source for those microbiota is the oropharynx and upper gastrointestinal tract [7]. Enterococcus and Candida species are predominantly responsible for infectious complications after anastomotic leakage. Prophylaxis with systemic antibiotic and antimycotic treatment is common in cancer patients after stem cell transplantation or in immunocompromised intensive care patients to prevent opportunistic infections [16]. In contrast, there are no consistent strategies to prevent anastomotic leakage after esophageal resection. In line with this strategy are the observations of Hochreiter and co-workers demonstrating that prophylactic prolonged antibiotic treatment does not reduce the rate of infectious complications after esophagectomy [17]. Several studies investigated the impact of selective decontamination of the esophagus prior to resection, which is an established concept in colorectal surgery [18–20]. With an appropriate choice of anti-infective agents, the burden of gram-negative bacteria and fungi is reduced while an anaerobic flora is maintained, resulting in strongly reduced microbe colonisation. Selective decontamination consists of oral application of Colistin, Tobramycin, and Amphotericin B [18]. Indeed, lower-level evidence suggests that perioperative decontamination of the esophagus results in a reduction of infectious complications, namely pneumonia and anastomotic leakage [18, 21]. However, no randomized-controlled data are available to support this strategy. Hence, the use of selective decontamination of the digestive tract is inconsistent among surgical centers performing esophageal surgery. Moreover, several other methods exist to prevent postoperative pneumonia. The usage of epidural analgesia and repetitive breathing exercises after surgery to improve chest wall motion and ventilatory capacity have been reported to reduce the risk of pulmonary complications after esophagectomy [22]. International guidelines further provide evidence that adequate lung re-expansion, assisted expectoration, aspiration pneumonia prevention, and nutritional support, reduce in less postoperative pulmonary infections and improved cardiac function [23]. Considering the price health care systems have to pay for infectious complications, more efforts are necessary to overcome this health burden.

Our study has several limitations that have to be mentioned.

First, it is a retrospective analysis with a relatively low number of patients. However, due to the analysis of three different cohorts in two different European countries, the external validity of this study is enhanced. Of note, we did not perform propensitiy score matching, hence, selection bias, potential confounders and interpretation of multivariate analysis might be hampered.

Second, only 40.5% of all patients included into the analysis were operated with minimally-invasive technique. As discussed above, minimally-invasive surgery is capable to reduce the rate of postoperative infectious complications after esophagectomy, especially pneumonia. Implementation of minimally-invasive techniques in esophageal cancer surgery was performed in the three study centers within the last two to three years. Therefore, the proportion of patients undergoing minimally-invasive esophagectomy during the study period was still rather low. However, the patient-dependent variables found in this study cannot be influenced using minimally-invasive surgical techniques. Third, using health system costs in the form of payments to hospitals in the DRG system is merely an estimation of the true cost of infectious complications after esophagectomy. However, these figures are easy to obtain and equivalent across hospitals in the given health care system.

Fourth, due to inconsistency of patients data, we were not able to include known risk factors for the occurrence of anastomotic leakage or infectious complications, such as diabetes, anemia, chronic kidney disease, and chronic obstructive pulmonary disease [6, 24].

## Conclusion

Infectious complications remain the most important challenge after esophagectomy. We present data including precise characterization of the subtype of microbiota associated with the occurrence of infectious complications after esophagectomy and its impact on health system costs. Early prevention to avoid its occurrence might improve the initial postoperative course of patients undergoing esophagectomy for esophageal cancer. This concept should be assessed in prospective studies.

## Supplementary Information

Below is the link to the electronic supplementary material.

Supplementary file1 Supplementary Table S1: Distribution of microbiota and fungi in bronchoalveolar lavage. (PDF 45 KB)

Supplementary file2 Supplementary Table S2: Distribution of microbiota and fungi in drainage fluid. (PDF 40 KB)

Supplementary file3 Supplementary Table S3: Distribution of microbiota and fungi in pleural effusion. (PDF 42 KB)

Supplementary file4 Supplementary Table S4: Distribution of microbiota and fungi in intraabdominal swab. (PDF 39 KB)

Supplementary file5 Supplementary Table S5: Distribution of microbiota and fungi in tracheal fluid. (PDF 42 KB)

Supplementary file6 Supplementary Table S6: Distribution of microbiota and fungi in wound swab. (PDF 42 KB)

Supplementary file7 Supplementary Table S7: Univariate analysis for variables associated with the occurrence of infectious complications (DOCX 13 KB)

## Data Availability

No datasets were generated or analysed during the current study.
