# Supplementary material for: Patterns of infectious complications and their implication on health system costs after esophagectomy for esophageal cancer: Real-world data from three European centers
Source: Langenbecks Arch Surg. 2025 Apr 22;410(1):138. doi: 10.1007/s00423-025-03709-5 (PMC12014832; doi:10.1007/s00423-025-03709-5)
Supplement: Supplementary file 1 — Supplementary file1 Supplementary Table S1: Distribution of microbiota and fungi in bronchoalveolar lavage. (PDF 45 KB) [file 423_2025_3709_MOESM1_ESM.pdf]

Bronchoalveolar lavage fluid	Species	Number of patients
	Candida albicans	11
	Staphylococcus epidermidis	4
	Greening streptococci	4
	Candida glabrata	3
	Enterococcus faecium	3
	Escherichia coli	3
	Klebsiella pneumoniae	3
	Serratia marcescens	3
	Staphylococcus aureus	2
	Achromobacter spp.	1
	Aspergillus fumigatus	1
	Citrobacter freundii	1
	Enterobacter aerogenes	1
	Enterococcus faecalis	1
	Haemophilus parahaemolyticus	1
	Haemophilus parainfluenzae	1
	Klebsiella oxytoca	1
	Staphylococcus aureus (Methicillin-resistent)	1
	Neisseria spp.	1
	Pseudomonas aeruginosa	1
