# Supplementary material for: Patterns of infectious complications and their implication on health system costs after esophagectomy for esophageal cancer: Real-world data from three European centers
Source: Langenbecks Arch Surg. 2025 Apr 22;410(1):138. doi: 10.1007/s00423-025-03709-5 (PMC12014832; doi:10.1007/s00423-025-03709-5)
Supplement: Supplementary file 2 — Supplementary file2 Supplementary Table S2: Distribution of microbiota and fungi in drainage fluid. (PDF 40 KB) [file 423_2025_3709_MOESM2_ESM.pdf]

Drainage fluid	Species	Number of patients
	Candida albicans	4
	Escherichia coli	3
	Enterobacter cloacae complex	2
	Enterococcus faecium	2
	Klebsiella oxytoca	2
	Enterococcus faecalis	1
	Morganella morganii	1
	Serratia marcescens	1
	Staphylococcus epidermidis	1
	Staphylococcus haemolyticus	1
	Stenotrophomonas maltophilia	1
	Streptococcus anginosus	1
	Streptococcus mitis	1
