# Supplementary material for: Patterns of infectious complications and their implication on health system costs after esophagectomy for esophageal cancer: Real-world data from three European centers
Source: Langenbecks Arch Surg. 2025 Apr 22;410(1):138. doi: 10.1007/s00423-025-03709-5 (PMC12014832; doi:10.1007/s00423-025-03709-5)
Supplement: Supplementary file 3 — Supplementary file3 Supplementary Table S3: Distribution of microbiota and fungi in pleural effusion. (PDF 42 KB) [file 423_2025_3709_MOESM3_ESM.pdf]

Pleural effusion	Species	Number of patients
	Enterococcus faecium	7
	Candida albicans	6
	Klebsiella pneumoniae	3
	Enterobacter cloacae complex	2
	Escherichia coli	2
	Prevotella melaninogenica	2
	Candida glabrata	1
	Candida kefyr	1
	Candida krusei	1
	Candida tropicalis	1
	Klebsiella oxytoca	1
	Staphylococcus aureus (Methicillin-resistent)	1
	Neisseria subflava	1
	Pediococcus pentosaceus	1
	Streptococcus mitis	1
	Veillonella atypica	1
