# Supplementary material for: Patterns of infectious complications and their implication on health system costs after esophagectomy for esophageal cancer: Real-world data from three European centers
Source: Langenbecks Arch Surg. 2025 Apr 22;410(1):138. doi: 10.1007/s00423-025-03709-5 (PMC12014832; doi:10.1007/s00423-025-03709-5)
Supplement: Supplementary file 4 — Supplementary file4 Supplementary Table S4: Distribution of microbiota and fungi in intraabdominal swab. (PDF 39 KB) [file 423_2025_3709_MOESM4_ESM.pdf]

Intraabdominal swab	Species	Number of patients
	Candida albicans	2
	Streptococcus mitis	2
	Enterobacter cloacae complex	1
	Escherichia coli	1
	Haemophilus parainfluenzae	1
	Prevotella melaninogenica	1
	Pseudomonas aeruginosa	1
	Staphylococcus epidermidis	1
