# Supplementary material for: Patterns of infectious complications and their implication on health system costs after esophagectomy for esophageal cancer: Real-world data from three European centers
Source: Langenbecks Arch Surg. 2025 Apr 22;410(1):138. doi: 10.1007/s00423-025-03709-5 (PMC12014832; doi:10.1007/s00423-025-03709-5)
Supplement: Supplementary file 5 — Supplementary file5 Supplementary Table S5: Distribution of microbiota and fungi in tracheal fluid. (PDF 42 KB) [file 423_2025_3709_MOESM5_ESM.pdf]

Tracheal fluid	Species	Number of patients
	Candida albicans	10
	Enterobacter cloacae complex	4
	Serratia marcescens	4
	Staphylococcus aureus	4
	Candida glabrata	3
	Enterococcus faecalis	3
	Escherichia coli	3
	Pseudomonas aeruginosa	3
	Stenotrophomonas maltophilia	3
	Enterococcus faecium	2
	Klebsiella pneumoniae	2
	Acinetobacter baumanii complex	1
	Candida dubliniensis	1
	Morganella morganii	1
	Neisseria species	1
	Staphylococcus epidermidis	1
	Greening streptococci	1
