# Supplementary material for: Patterns of infectious complications and their implication on health system costs after esophagectomy for esophageal cancer: Real-world data from three European centers
Source: Langenbecks Arch Surg. 2025 Apr 22;410(1):138. doi: 10.1007/s00423-025-03709-5 (PMC12014832; doi:10.1007/s00423-025-03709-5)
Supplement: Supplementary file 6 — Supplementary file6 Supplementary Table S6: Distribution of microbiota and fungi in wound swab. (PDF 42 KB) [file 423_2025_3709_MOESM6_ESM.pdf]

Wound swab	Species	Number of infection cases
	Candida albicans	8
	Enterococcus faecium	4
	Pseudomonas aeruginosa	3
	Enterococcus faecalis	3
	Candida glabrata	3
	Escherichia coli	2
	Candida dubliniensis	2
	Enterobacter cloacae complex	2
	Streptococcus mitis	2
	Staphylococcus aureus	1
	Morganella morganii	1
	Staphylococcus haemolyticus	1
	Haemophilus parainfluenzae	1
	Prevotella melaninogenica	1
	Proteus mirabilis	1
	Staphylococcus epidermidis	1
	Staphylococcus constellatum	1
