# Supplementary material for: Patterns of infectious complications and their implication on health system costs after esophagectomy for esophageal cancer: Real-world data from three European centers
Source: Langenbecks Arch Surg. 2025 Apr 22;410(1):138. doi: 10.1007/s00423-025-03709-5 (PMC12014832; doi:10.1007/s00423-025-03709-5)
Supplement: Supplementary file 7 — Supplementary file7 Supplementary Table S7: Univariate analysis for variables associated with the occurrence of infectious complications (DOCX 13 KB) [file 423_2025_3709_MOESM7_ESM.docx]

| **ANOVA** | **(p value)** |
| --- | --- |
| **Gender** | 0.962 |
| **ASA Score** | 0.012 |
| **Age** | 0.081 |
| **BMI** | 0.106 |
| **Obesity** | 0.765 |
| **Reflux** | 0.482 |
| **Barrett dysplasia** | 0.684 |
| **Smoking** | 0.831 |
| **Alcohol abuse** | 0.107 |
